# One-pot encapsulation of lactate dehydrogenase and Fe_3_O_4_ nanoparticles into a metal–organic framework: A novel magnetic recyclable biocatalyst for the synthesis of D-phenyllactic acid

**DOI:** 10.3389/fbioe.2022.1124450

**Published:** 2023-01-09

**Authors:** Xiaolong Sun, Jiahuan Hu, Yifeng Wang, Xi Luo, He Huang, Yongqian Fu

**Affiliations:** ^1^ State Key Laboratory of Material-Oriented Chemical Engineering, School of Pharmaceutical Sciences, Nanjing Tech University, Nanjing, China; ^2^ Taizhou Key Laboratory of Biomass Functional Materials Development and Application, Taizhou University, Taizhou, China; ^3^ School of Food Science and Pharmaceutical Engineering, Nanjing Normal University, Nanjing, China

**Keywords:** biocatalyst, d-phenyllactic acid, lactate dehydrogenase, LDH/MNPs@MAF-7, magnetic nanoparticles

## Abstract

The main challenges in bio-catalysis of d-phenyllactic acid (D-PLA) are poor tolerance of lactate dehydrogenase (LDH) to harsh environmental conditions and inability to recycle the catalyst. A novel magnetic framework composite was prepared as solid support for the immobilization of enzymes *via* one-pot encapsulation in this study. LDH/MNPs@MAF-7 was synthesized by the one-pot encapsulation of both LDH and magnetic nanoparticles (MNPs) in MAF-7. The LDH/MNPs@MAF-7 showed stable biological activity for the efficient biosynthesis of D-PLA. The structure and morphology of LDH/MNPs@MAF-7 were systematically characterized by SEM, FT-IR, XRD, VSM, XPS, TGA and N_2_ sorption. These indicated that LDH/MNPs@MAF-7 was successfully synthesized, exhibiting enhanced resistance to acid and alkali, temperature and organic solvents. Furthermore, the bio-catalyst could be separated easily using a magnet, and the reusability was once considerably expanded with 80% of enzyme activity last after eight rounds of recycling. Therefore, LDH/MNPs@MAF-7 could be used as a potential biocatalyst for the biosynthesis of D-PLA due to its good stability and recovery properties.

## 1 Introduction

D‐phenyllactic acid (D-PLA) has been validated to have inhibitory effects against yeast and a huge variety of molds, together with some mycotoxigenic species (Lavermicocca et al., 2000; Lavermicocca et al., 2003), which gives it great application potential in the food and medicine related fields. Recently, D-PLA has attracted more attention due to the raised food safety awareness (Wu et al., 2021), but the yield of D-PLA that can be obtained through direct microbial fermentation remains low. Nowadays, lactate dehydrogenase (LDH) is expressed in *Escherichia coli* (*E. coli*) to produce D-PLA using phenylpyruvic acid (PPA) as substrate (Luo et al., 2020b). However, enzymes are limited used in industrial applications for various reasons (Torkzadeh-Mahani et al., 2020), including high cost, low substrate or product tolerance, poor stability in harsh environments, and poor reusability, which limit their practical applications (Sirisha et al., 2016; Du et al., 2022). An effective and common way to extend the tolerance of enzymes is the use of immobilization technology (Chen et al., 2019). In a study by Wang K. et al. (2022), a nascent α-amylase nano-biocatalytic system was constructed using the natural nanostructured mineral montmorillonite, and the prepared immobilized α-amylase exhibited greater stability and higher catalytic activity over a harsh environment. Similarly, Ahmed et al. (2018) successfully fabricated Cellulase@UiO-66-NH_2_, which exhibited a significant improvement in tolerance of temperature, pH, reusability and lifetime. Immobilization is an effective tool for enzyme preservation and continuous operation (Siar et al., 2017; Alagoz et al., 2021; Wei et al., 2021).

Metal-organic frameworks (MOFs) have attracted increasing attention as carriers in immobilization systems of enzyme due to their excellent features such as high surface area and favorable biocompatibility, well-defined pore and crystal structure (Du et al., 2022). Nadar and Rathod (2020) encapsulated a lipase inside a ZIF-8 using a biomineralization method. The synthesized immobilized lipase-proline showed a 135% increase in catalytic activity and 4-fold improvement of thermal stability compared to the free enzyme. Wei et al. (2021) first constructed renewable and magnetic MOFs (immobilized β-glucuronidase), and the immobilized enzymes exhibited greater stability over a wide range of temperatures and pH. Liang et al. (2021) noted that immobilized enzymes to hydrophilic ZIF-90 or MAF-7 could maintain enzyme activity, even when exposed to harsh reaction environment. Due to the features mentioned above, encapsulating enzymes in MOFs is an increasingly popular method for enzyme immobilization and protection (Tranchemontagne et al., 2009; Hirai et al., 2014). Especially, zeolitic metal-azolate framework-7 (MAF-7) is one of the most concerned MOFs for enzyme immobilization due to its high stability.

For the sake of simplicity in the downstream separation and recovery operations of enzymatic catalyzed processes, magnetic nanoparticles (MNPs) such as Fe_3_O_4_, have been co-captured in MOFs together with enzyme. Cao et al. (2022) suggested that the presence of Fe_3_O_4_ nanoparticles could influence the electron spin state of intermediates and promote enzyme activation through a magnetic induction effect during the reaction process. They immobilized the laccase on magnetic framework composite, and the removal rate of 2,4-dichlorophenol is still more than 80% after nine times of recycling. The prepared magnetic framework composites (MFCs) can be easily recovered by magnet with minimal losses, and the recovered lysozyme or α-amylase/MNP@MOF can often retain stable activity (Li et al., 2020).

To our best knowledge, there are no studies on the biosynthesis of D-PLA using magnetic framework composite, which encapsulated both LDH and MNPs. Therefore, in this work, we attempted to synthesis a novel magnetic framework composite for immobilization of LDH as a biocatalyst to catalyze the synthesis of PLA. On this basis, we tried to characterize the structure and morphology of the MFCs using FTIR, XRD, etc. Meanwhile, in order to evaluate the stability of this magnetic catalyst, the association between reaction environment (temperature, pH, solvent) and enzyme activity was also investigated. This modification method could probably provide viable ideas for the construction and performance optimization of enzyme immobilization composite in the future.

## 2 Material and methods

### 2.1 Materials

3-methyl-1,2,4-triazole (Adamas-beta, Hmtz), zinc nitrate hexahydrate (Sinopharm Chemical Reagent Co., Ltd., Zn (NO_3_)_2_·6H_2_O), NH_3_·H_2_O (25%, Macklin), sodium phenylpyruvate (PPA, Aladdin), Fe_3_O_4_ (Aladdin), Bradford protein detection kit (TaKaRa). None of the chemicals were further purified before usage.

### 2.2 Synthesis of LDH/MNPs@MAF-7

The expression and purification of D-LDH (hereinafter referred to as LDH) refer to the previous research methods of our laboratory (Luo et al., 2020a). The LDH expression strain was grown and induced expression in Luria-Bertani (LB) medium containing 50 μg mL^−1^ kanamycin. Cells were collected by centrifugation and LDH crude enzyme solution was obtained from lysed cells. The LDH crude enzyme was purified using Ni^2+^-nitrilotriacetic acid column (1.6 cm × 10 cm, BioRad, United States). And then dialyzed overnight against 20 mM potassium phosphate buffer (pH 8.0) to obtain purified LDH. See support information for specific experimental steps (S 1.1 Expression and purification of d-lactate dehydrogenase).

LDH/MNPs@MAF-7 was synthesized in an aqueous system (total volume was 4 ml) comprising Zn (NO_3_)_2_·6H_2_O (40 mM), Hmtz (120 mM), Fe_3_O_4_ (20 mM), NH_3_·H_2_O (10%, 60 μL) and the indicated concentration of LDH at 25°C under stirring (200 rpm) for 24 h. The obtained material was recovered with magnets, washed thoroughly in distilled water and repeated three times to remove loosely adsorbed LDH.

### 2.3 Characterization of LDH/MNPs@MAF-7

#### 2.3.1 Scanning electron microscopy (SEM) analysis

The SEM observations of samples were performed on scanning electron microscope (Hitachi S-4800, Japan) with an acceleration voltage set to 30 kV. Before the SEM analysis, the samples are fixed on a stainless-steel frame and sprayed with gold using an ion sprayer. And the elemental composition was also measured by Hitachi S-4800 equipped with energy-disperse spectrometer (EDS) attachment.

#### 2.3.2 FT-IR analysis

The FT-IR spectra of samples were determined by Fourier transform infrared spectrometer (Nicolet 5,700, Thermo Nicolet, United States of America). Each sample was mixed with 200 mg of anhydrous KBr and pressed into pellets, which were scanned and analyzed 16 times on the FT-IR spectroscope to record the spectra in the frequency range of 400–4,000 cm^−1^ at a resolution of 4 cm^−1^.

#### 2.3.3 X-ray diffraction (XRD) analysis

XRD patterns of MAF-7, MNPs@MAF-7, and LDH/MNPs@MAF-7 were recorded using D8 Advance diffractometer (Bruker, Germany) under the following operating conditions: 40 kV and 40 mA with acceptance slot at 0.1 mm and Cu Kα radiation at *λ* = 0.15405 nm. The relative intensity was recorded in the scattering range of 5–80°2θ at a step of 10°min.

#### 2.3.4 Vibrating-sample magnetometer (VSM) analysis

The magnetic properties of samples were analyzed with a PPMS-9T vibrating sample magnetometer (Quantum Design, United States) at room temperature. The range of varying magnetic field during scanning is from −20000 to 20,000 Oe. The default sample vibration frequency for the test was 40 Hz.

The VSM curves were measured at room temperature under a varying magnetic field from −20000 to 20,000 Oe on a PPMS-9T vibrating sample magnetometer (Quantum Design, United States).

#### 2.3.5 X-ray photoelectron spectroscopy (XPS) analysis

The XPS measurement was measured with ESCALAB 250Xi spectrometer (Thermo Scientific, United States of America). The test passing-energy was 50 eV, the step length was 0.05 eV, and the combined Energy standard C 1s = 284.80 eV was used for charge correction.

#### 2.3.6 Thermogravimetric analysis (TGA)

TGA data were collected on a synchronous thermal analyzer (STA) (TGA/DSC, Mettler Toledo, Switzerland). Samples were heated from room temperature to 900°C at a rate of 10°C/min. Samples were heated at a constant airflow rate.

#### 2.3.7 Nitrogen adsorption analysis

Nitrogen adsorption and desorption isotherms were recorded on an ASAP-2020-HD88 (Micromeritics, United States) surface characterization analyzer. Approximately 20 mg sample was degassed under dynamic vacuum (12°h, 105°C). After that, the analytical experiments were performed at 277 K.

### 2.4 Enzyme activity measurement of LDH/MNPs@MAF-7

The enzyme activity was measured by measuring the concentration of D-PLA catalyzed, using PPA (20 mM) and NADH (20 mM) as substrate. HPLC was used for detecting the concentration of D-PLA (Luo et al., 2020a). Specific test conditions refer to the laboratory published literature (Wang Y. et al., 2022). The standard curve of enzyme activity assay is shown in Supplementary Figure S3.

### 2.5 Stability analysis of LDH/MNPs@MAF-7

#### 2.5.1 Temperature stability

LDH/MNPs@MAF-7 was incubated at 40°–80°C for 20 min in tris-HCl buffer, and the residual activity was compared with the control incubated at 30°C.

#### 2.5.2 pH stability

LDH/MNPs@MAF-7 has incubated buffers with the indicated pH values (4–10) for 20 min. The treated materials were recovered with magnets and washed thoroughly with distilled water.

#### 2.5.3 Organic solvent stability

LDH/MNPs@MAF-7 was incubated in the presence of different organic solvents, including dimethyl sulfoxide (DMSO), N, N-dimethylformamide (DMF), and dichloromethane (DCM) for 15 min. The treated materials were collected with magnets and washed thoroughly with distilled water.

### 2.6 Reusability

LDH/MNPs@MAF-7 was repeated eight times and the residual activity was measured after each use. After each cycle, it was collected with magnets, then added with a new substrate solution (20 mM PPA and NADH) to start a new catalytic reaction.

## 3 Results and discussion

### 3.1 One-pot encapsulation strategy for immobilizing LDH

**FIGURE 1:**
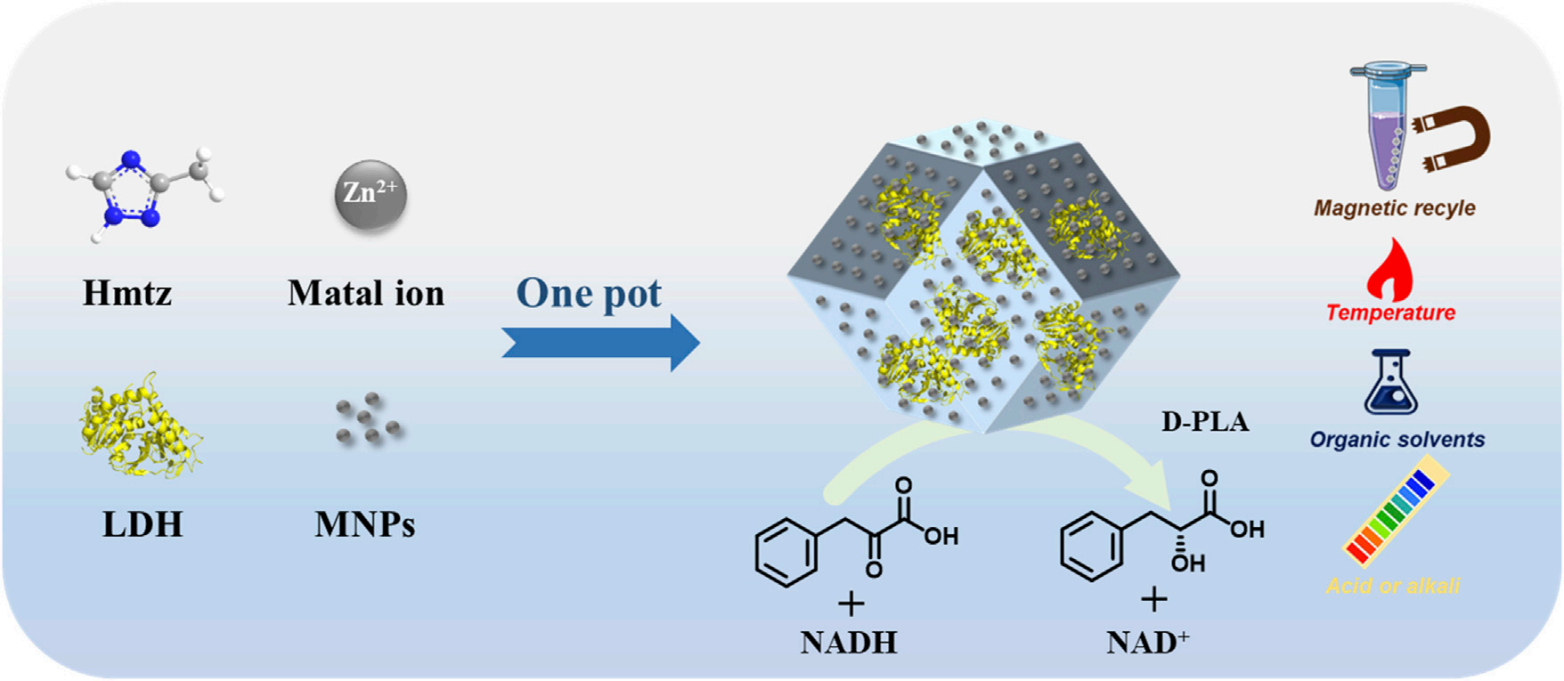
The synthesis of the biocatalyst LDH/MNPs@MAF-7 and the catalytic activity evaluation.

By adopting a one-pot encapsulation strategy, we encapsulated the LDH and MNPs into MAF-7 frame. The performances of the LDH/MNPs@MAF-7 composite were evaluated under various conditions using the preparation of D-PLA as a model reaction (Figure 1).

### 3.2 SEM analysis

**FIGURE 2:**
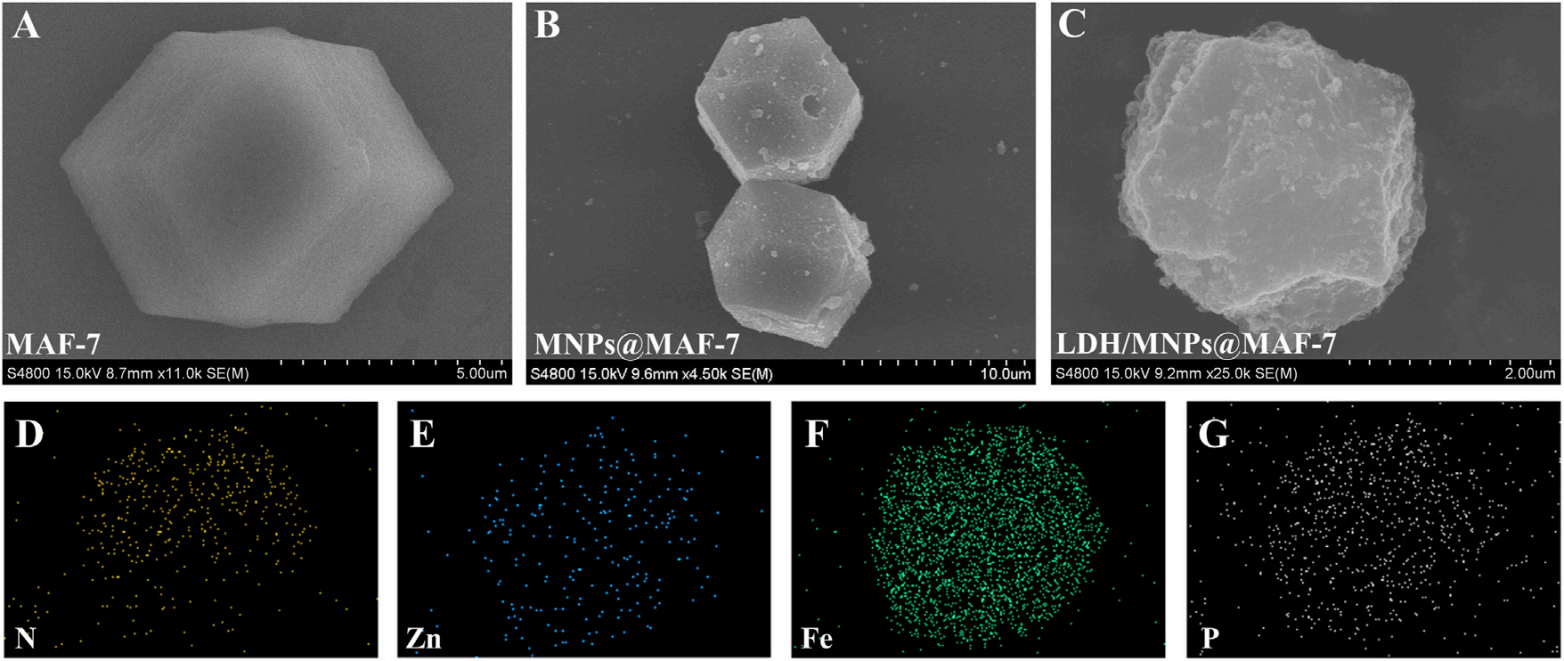
SEM spectra of **(A)** MAF-7 **(B)** MNPs@MAF-7, and **(C)** LDH/MNPs@MAF-7; elemental mapping **(D–G)** of LDH/MNPs@MAF-7.

The ultrastructural characteristics of MAF-7 (Figure 2A), MNPs@MAF-7 (Figure 2B), and LDH/MNPs@MAF-7 (Figure 2C) were observed *via* SEM. The MAF-7 particles displayed a typical dodecahedral structure with a diameter of approximately 2 –10 μm.

As shown in Figure 2C, LDH/MNPs@MAF-7 exhibited a roughly spherical shape. This may be due to a number of the enzyme is encapsulated and some is exposed. According to the SEM images, the particle sizes of LDH/MNPs@MAF-7 were smaller than those of MAF-7 and MNPs@MAF-7, which might be due to the enzyme immobilization. Ji et al. (2021) observed a similar phenomenon when encapsulating lipase and Fe_3_O_4_ in ZIF-8. Additionally, elemental mapping (Figures 2D–G) demonstrated that the sample contains nitrogen, zinc, iron, and phosphorus, which is in good agreement with the expected chemical composition of LDH/MNPs@MAF-7.

### 3.3 FT-IR analysis

**FIGURE 3:**
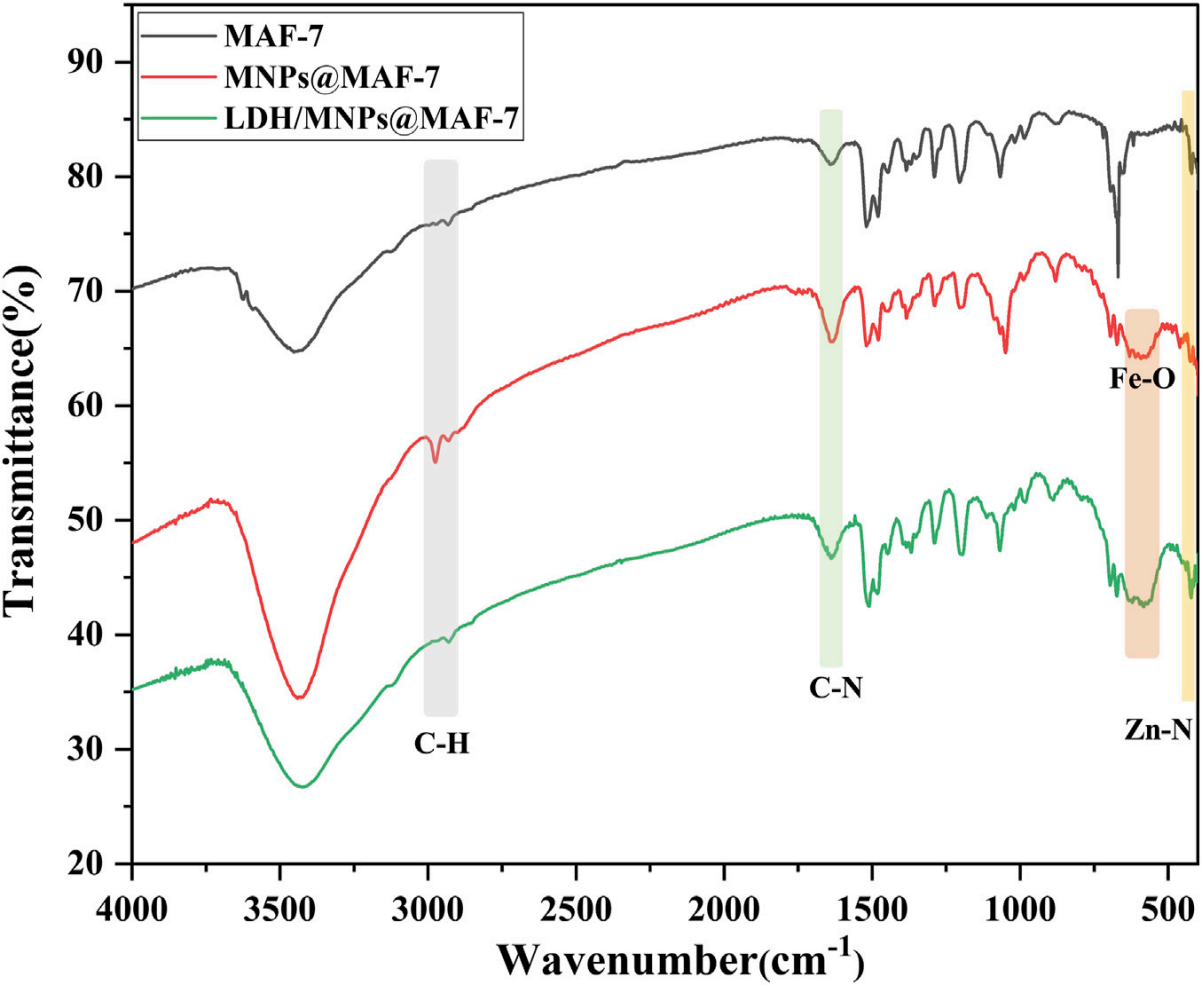
FT-IR spectra of MAF-7, MNPs@MAF-7, and LDH/MNPs@MAF-7.

The FT-IR bands corresponding to the different functional groups of MAF-7, MNPs@MAF-7, and LDH/MNPs@MAF-7 are indicated in Figure 3. In the FT-IR spectrum of MAF-7, the strong peaks around 424, 1,640 and 2,930 cm^−1^ could correspond to the stretching vibrations of Zn-N, C-N, and imidazole C-H, respectively (Tian et al., 2020; Wang Q. et al., 2022). Several bands were observed in the broad region of 2,500–3,500 cm^−1^, which corresponded to the stretching vibrations of N-H, O-H, and C-H. In the FT-IR spectra of Fe_3_O_4_ particles, a well-defined band near 600 cm^−1^ indicated the presence of Fe-O bonds. The FI-IR spectrum of MAF-7 was consistent with that of MNPs@MAF-7 and LDH/MNPs@MAF-7, barring the peak around 600 cm^−1^ (Fe-O). Thus, the FT-IR spectra indicated that both LDH and Fe_3_O_4_ were successfully immobilized in the MAF-7.

### 3.4 XRD analysis

**FIGURE 4:**
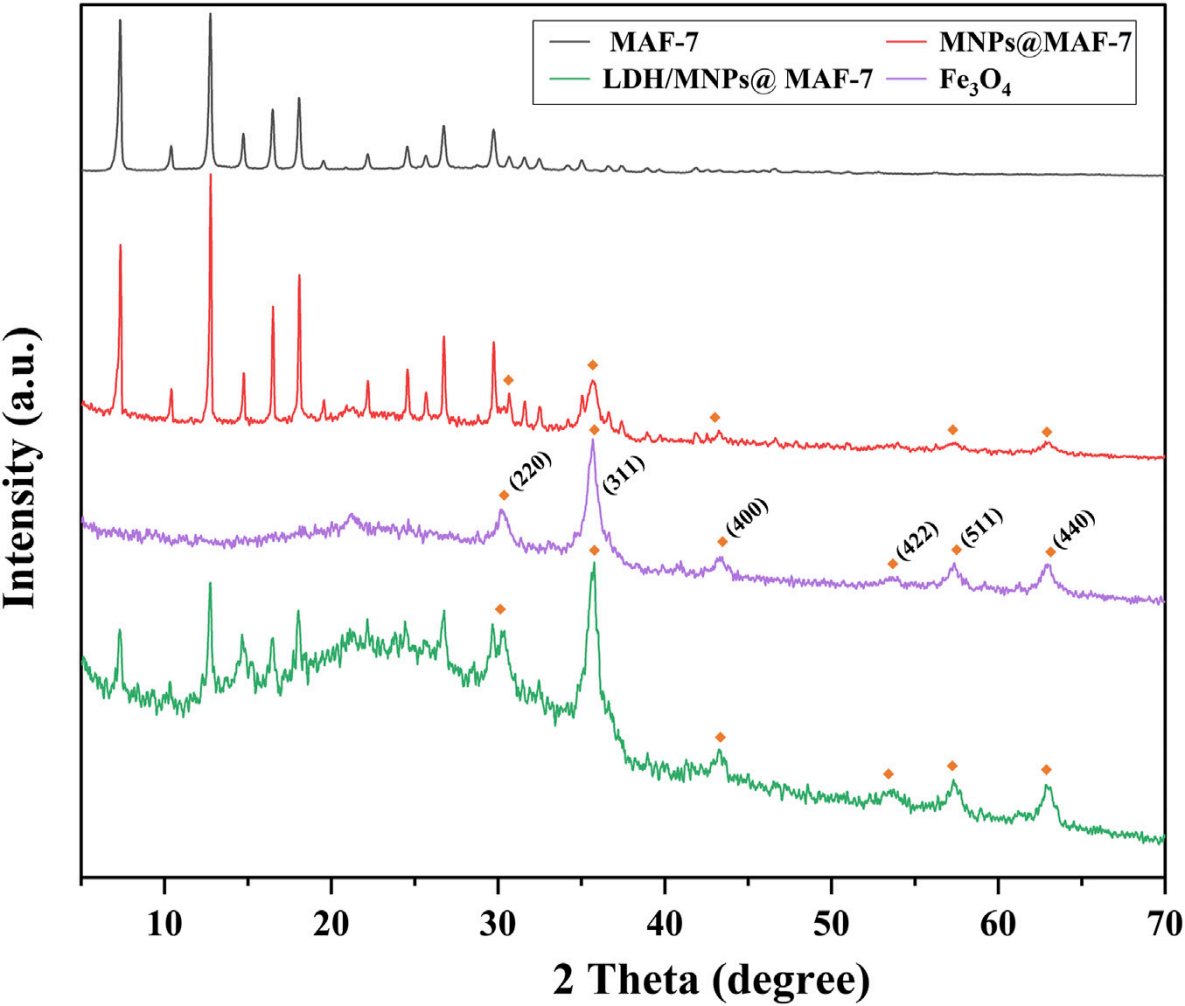
XRD patterns of MAF-7, MNPs@MAF-7, and LDH/MNPs@MAF-7.

XRD analysis was performed using pure MAF-7, Fe_3_O_4_, MNPs@MAF-7, and LDH/MNPs@MAF-7 (Figure 4). Compare to MAF-7, it showed a slightly decreased in the intensity of the characteristic diffraction peaks of LDH/MNPs@MAF-7. It is possibly because that part of MAF-7 sites is occupied by enzyme molecules, which partly disorganized the crystalline structure of the MOF. In addition, the XRD pattern of Fe_3_O_4_ nanoparticles showed six characteristic peaks at 2θ values of 30.26°, 35.74°, 43.22°, 53.32°, 57.24° and 62.8°, respectively corresponding to planes (220) (311) (400) (422) (511) and (440) of the face-centered cubic spinel Fe_3_O_4_ structure (Nosike et al., 2020). The XRD patterns collected for LDH/MNPs@MAF-7 indicated that the crystal structure of MAF-7 was not influenced by enzyme and magnetic nanoparticles immobilization.

### 3.5 Magnetic properties

**FIGURE 5:**
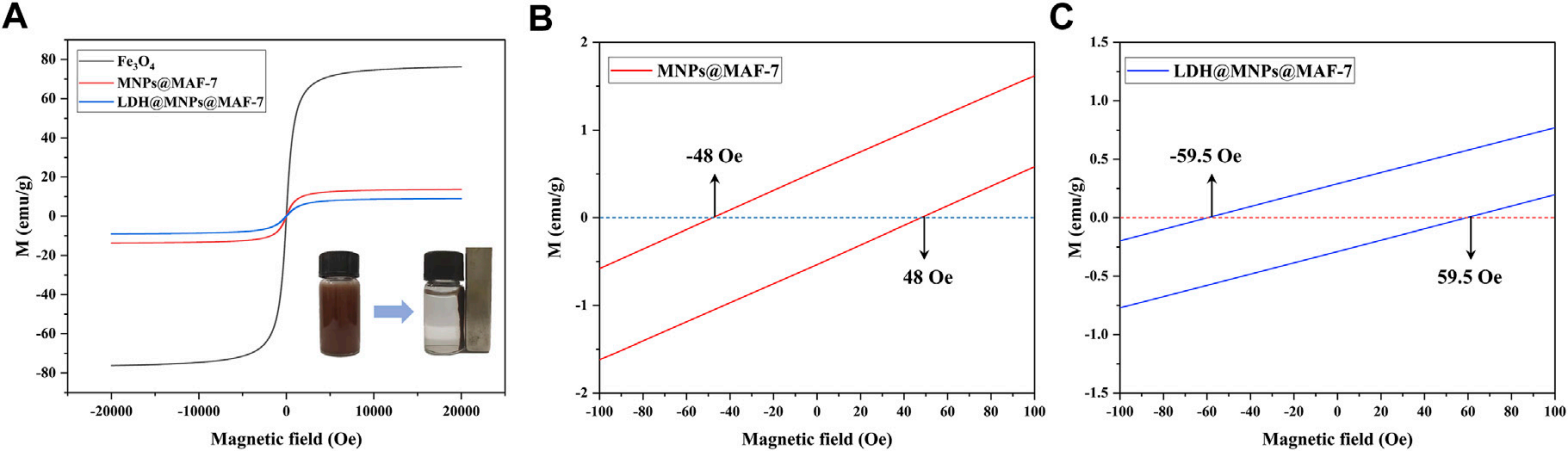
**(A)** Magnetic properties of Fe_3_O_4_, MNPs@MAF-7 and LDH/MNPs@MAF-7 at 300 K (Right inset: separation of the LDH/MNPs@MAF-7 particles from aqueous solution using an external magnetic field) **(B)** The magnified curves of MNPs@MAF-7 between -100 and 100 Oe **(C)** The magnified curves of LDH/MNPs@MAF-7 between -100 and 100 Oe.

The VSM with the corresponding field of −20 KOe ∼20 KOe was employed to study the magnetic properties of prepared MNPs@MAF-7 and LDH/MNPs@MAF-7 at 300 K. As shown in Figure 5, the appeared magnetism increased with the external magnetic field until the saturation magnetization of the MNPs@MAF-7 and LDH/MNPs@MAF-7 was reached at Ms = 13.65 and 8.98 emu/g. This value was much smaller than that of bulk Fe_3_O_4_ (76.2 emu/g), and the magnetization of Fe_3_O_4_ was basically consistent with that reported in the literature (81.3 emu/g) (Ge et al., 2020). This indicates that the MAF-7 and LDH shell does not contribute to the magnetization, resulting in a smaller magnetic moment per unit mass. However, a strong response to an external magnetic field was still observed (inset of Figure 5A). After the significant amplification, the coercivity (defined as the field magnitude necessary to obtain M = 0) was as low as 48 and 59.5 Oe, respectively, indicating that the microspheres can be easily dispersed in the absence of an external magnetic field. This directly demonstrates that the LDH/MNPs@MAF-7 possess strong magnetic properties. It will be easy and efficient to separate LDH/MNPs@MAF-7 particles from the reaction system.

### 3.6 XPS analysis

**FIGURE 6:**
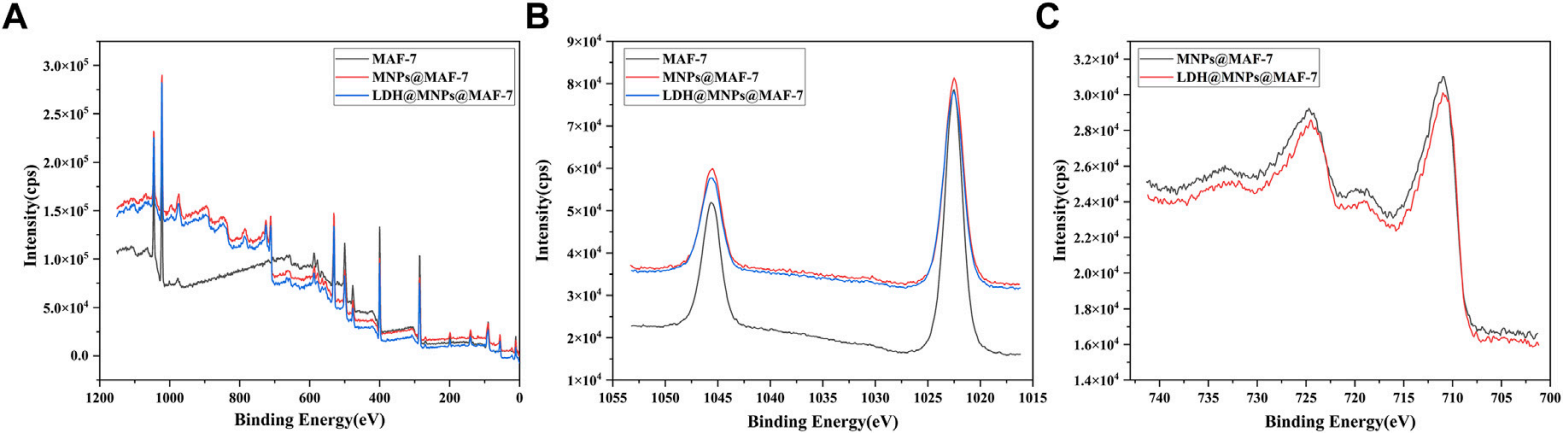
**(A)** XPS spectra of MAF-7, MNPs@MAF-7 and LDH/MNPs@MAF-7 **(B)** XPS spectra of Zn 2p in MAF-7, MNPs@MAF-7 and LDH/MNPs@MAF-7; and **(C)** XPS spectra of Fe 2p in MNPs@MAF-7 and LDH/MNPs@MAF-7.

The MAF-7, MNPs@MAF-7 and LDH/MNPs@MAF-7 were analyzed by X-ray photoelectron spectroscopy to investigate the composition as well as the chemical state. In Figure 6A, similar coordination of Zn and N was present in MNPs@MAF-7 and LDH/MNPs@MAF-7 as in MAF-7. The peak at 1,022.48 eV corresponded to Zn 2p peak (Figure 6B), slightly higher than the standard binding energy of Zn^2+^ (1,022 eV), which also indicated that the chemical environment of Zn is dominated by N-coordination (Wang J. et al., 2020). As illustrated in Figure 6C, there are two characteristic peaks at 710.92 and 724.68 eV, which were interpreted as the Fe 2p peaks of Fe_3_O_4_. As demonstrated in Supplementary Table S2, we calculated the chemical composition of the elements (C, N, Zn, P, Fe) through the integration of the peak areas. The existence of Fe and P suggested the successful encapsulation of MNP and LDH.

### 3.7 TGA analysis

**FIGURE 7:**
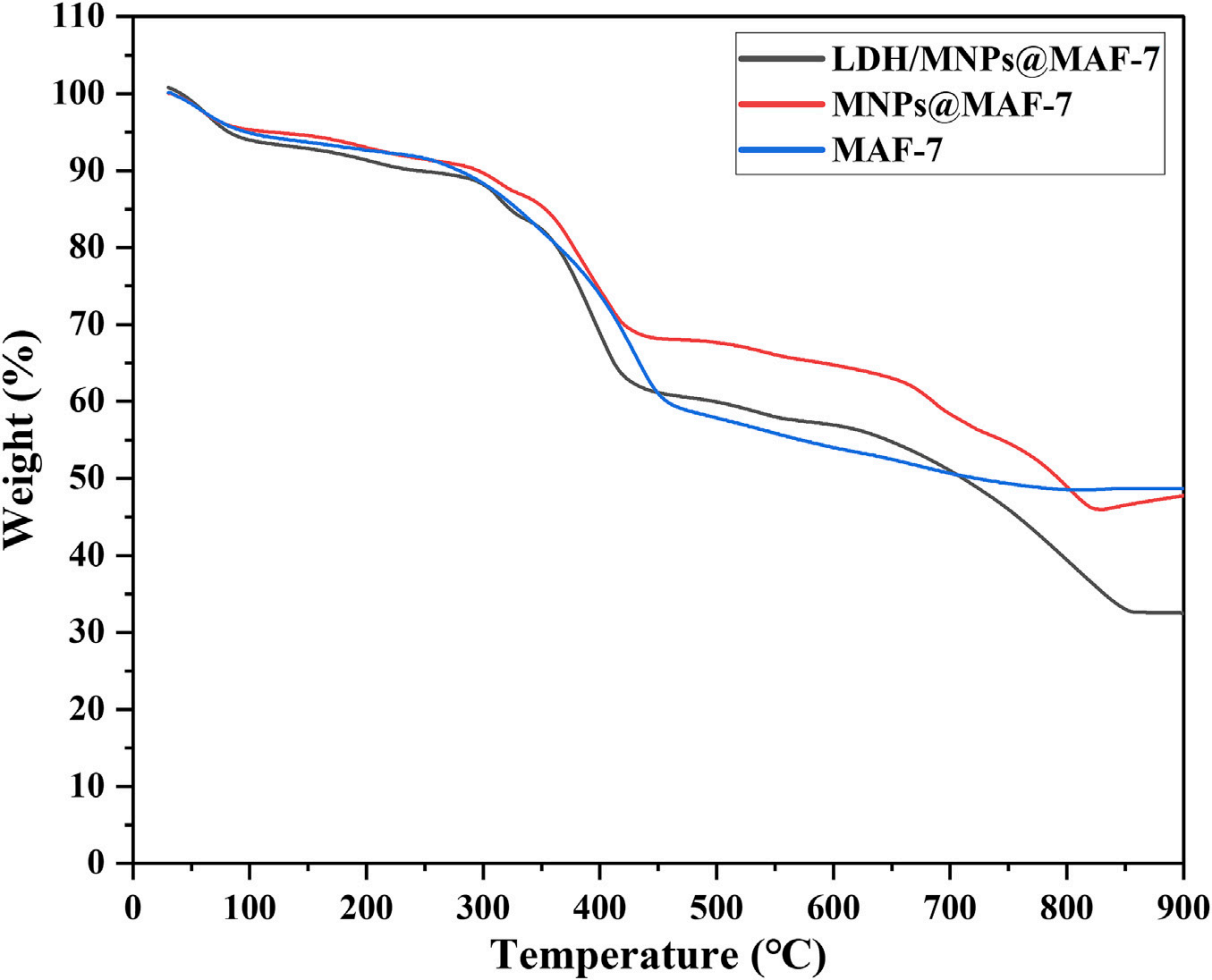
Thermogravimetric analysis (TGA) of MAF-7, MNPs@MAF-7 and LDH/MNPs@MAF-7.

The TGA curves exhibited two platforms, meaning two obvious mass loss phases (Figure 7). The initial weight loss phases (start from 30 to 400°C) may corresponded to the removal of water molecules and unreacted materials from the pores (Wang Y. et al., 2022).

The deformation of frame probably accounted for the second mass loss phase (from 400 to 850°C), which was more conspicuous in LDH/MNPs@MAF-7 (Wang L. et al., 2020). After heating to 850°C, the remaining weight of LDH/MNPs@MAF-7 and MNPs@MAF-7 was 32.6 and 45.7%, respectively. This can be explained by the loss of protein, which illustrates that LDH is successfully encapsulated in MAF-7 framework (Shieh et al., 2013; Shieh et al., 2015). According to the results of TGA, the loading efficiency of the enzyme in LDH/MNPs@MAF-7 was 12.1%.

### 3.8 Nitrogen adsorption analysis

**FIGURE 8:**
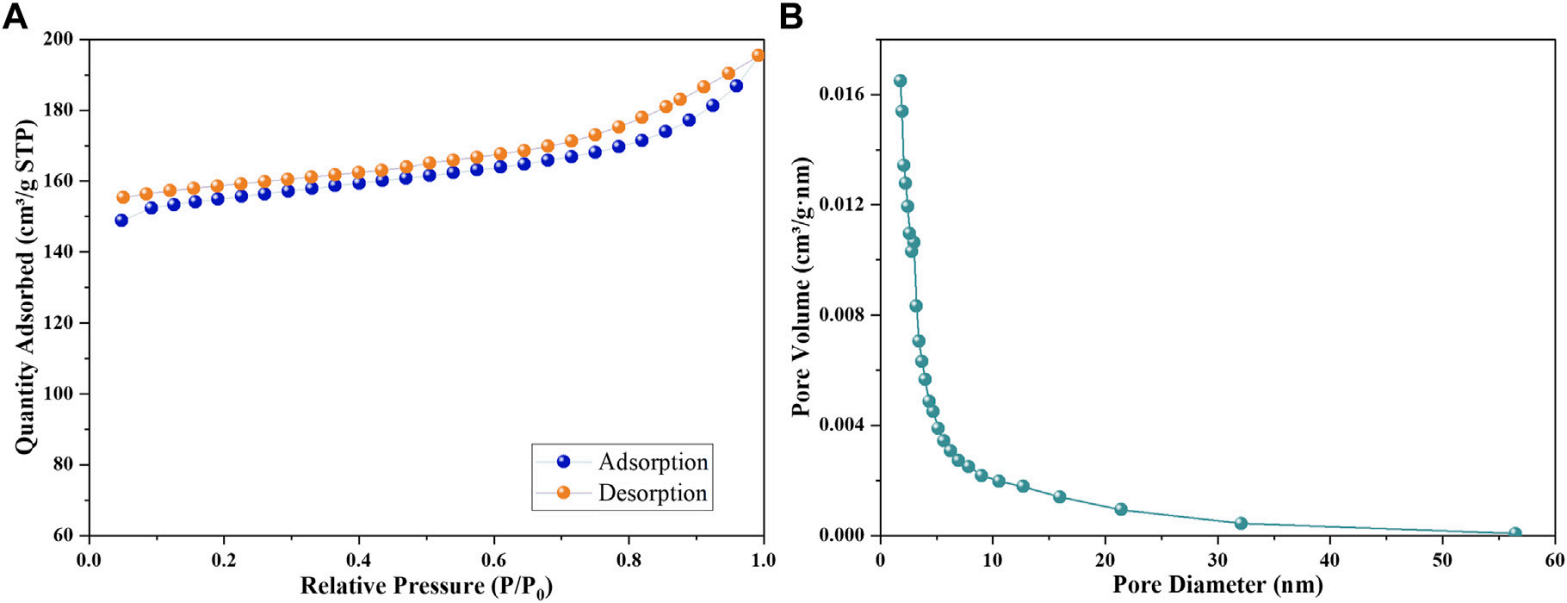
**(A)** N_2_ adsorption-desorption isotherms of LDH/MNPs@MAF-7 **(B)** The pore size distribution of LDH/MNPs@MAF-7.

In order to investigate the BET surface area and pore structure of MAF-7 (Figure 8A), MNPs@MAF-7 (Figure 8B) and LDH/MNPs@MAF-7 (Figure 8C), the N2 absorption and desorption experiments were conducted. The isotherms were categorized as a type-Ⅱ hysteresis loop attributed to the microporous structure of LDH/MNPs@MAF-7, which is similar to the isotherms of MAF-7. The specific surface area of LDH/MNPs@MAF-7 was ∼511 m^2^/g, which was smaller than MNPs@MAF-7 (∼755 m^2^/g) and MAF-7 (∼1,115 m^2^/g), due to the encapsulation of LDH and MNPs (Schejn et al., 2015; Wang L. et al., 2020). Further comparison of the pore diameters of LDH/MNPs@MAF-7, MNPs@MAF-7 and MAF-7 (Figure 8D) suggested that LDH/MNPs@MAF-7 and MNPs@MAF-7 had similar pore structure with MAF-7, illuminating good structural preservation during the encapsulation process.

### 3.9 Evaluation of stability and recyclability

**FIGURE 9:**
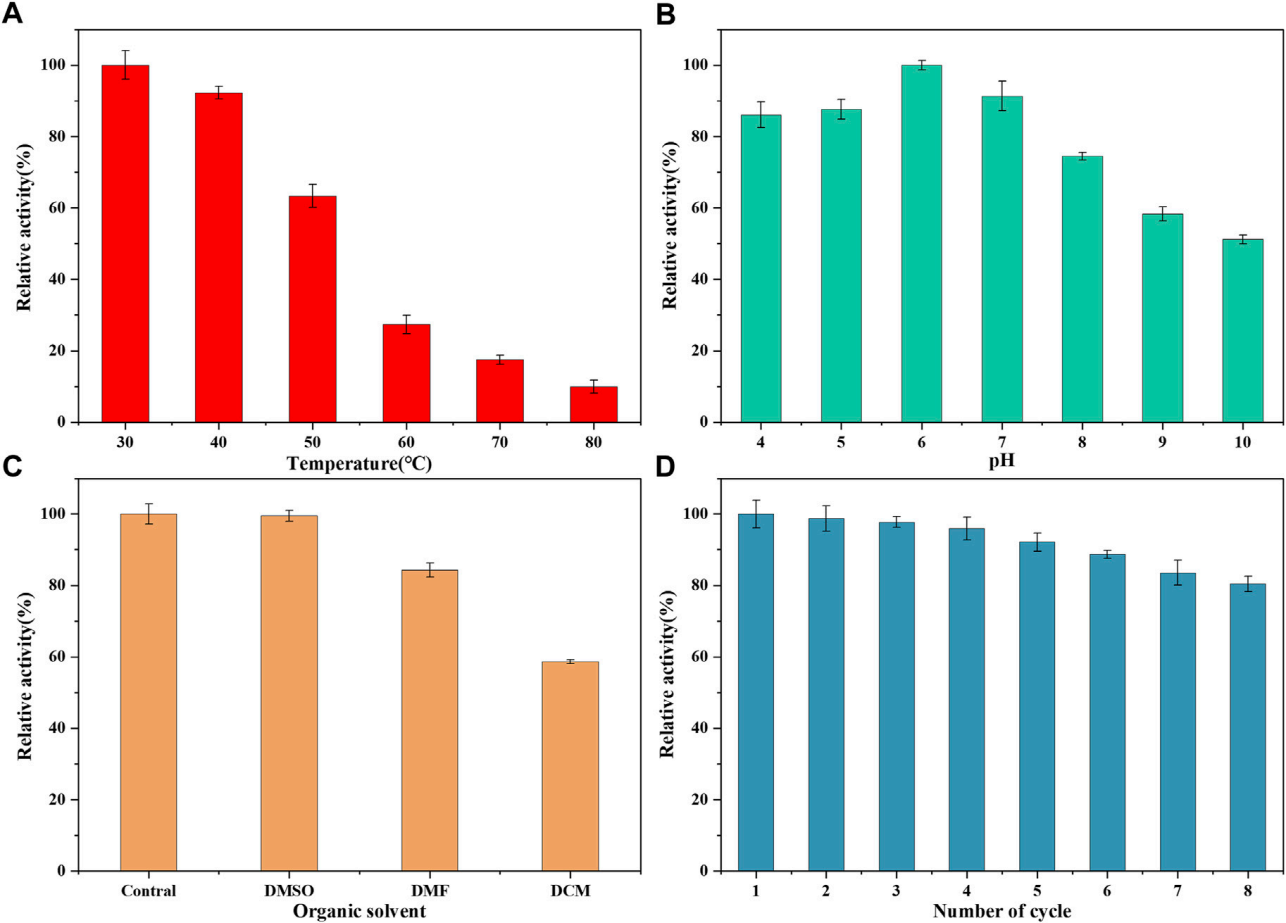
**(A)** Activity of LDH/MNPs@MAF-7 after incubation at the indicated temperatures for 20 min **(B)** Activity of LDH/MNPs@MAF-7 after incubation at different pH values for 20 min **(C)** Organic solvent stability of LDH/MNPs@MAF-7 **(D)** Reusability of LDH/MNPs@MAF-7 up to the 8th reuse cycle.

The enzymatic properties of LDH/MNPs@MAF-7 were investigated using pyruvic acid as the substrate. To evaluate the ability of MAF-7 to protect LDH at high temperatures, LDH/MNPs@MAF-7 was incubated at 30°C–80°C for 20 min. As displayed in Figure 9A, the relative enzyme activity retention rate of LDH/MNPs@MAF-7 decreased with increasing temperature, but the catalyst retained more than 10% of the maximum activity even at 80°C. By contrast, the free LDH was practically completely inactivated at 80°C. The enhanced thermal stability could be illustrated that LDH was protected by the rigid structure of MAF-7. This can minimize the unfolding of the enzyme (Ji et al., 2022).

To evaluate the influence of pH on the catalytic activity of LDH, the catalyst was incubated in tris-HCl buffers with different pH (4–10). As shown in Figure 9B, LDH/MNPs@MAF-7 demonstrated better tolerance under acidic conditions, and the activity reached the maximum at pH 6. This indicates that the three-dimensional structure of MAF-7 provided a microenvironment, thus helping the LDH retain its activity and preventing the subunits dissociation (Li et al., 2018; Ji et al., 2022).

The solubility of PPA is very poor, and it needs to be solubilized with organic solvents. Thus, LDH/MNPs@MAF-7-catalyzed reactions have been assessed in the presence of a variety of organic solvents (DMSO, DMF, DCM) (Figure 9C). LDH/MNPs@MAF-7 showed excellent tolerance to organic solvents (>58% residual activity). It has been reported that MAF-7 forms a barrier, which effectively reduced exposure to enzymes and organic solvents (Liang et al., 2015; Nadar and Rathod, 2019). The results indicated that the LDH/MNPs@MAF-7 was suitable for the biosynthesis of PLA.

Finally, the reusability of LDH/MNPs@MAF-7 was assessed as an important performance indicator for actual applications. After each cycle, the LDH/MNPs@MAF-7 were recycled and washed completely, then repeated the experiment of enzyme activity assessment. As illustrated in Figure 9D, the residual activity remained above 80% after eight successive cycles. The results demonstrated that LDH/MNPs@MAF-7 had a certain technical dominance in the D-PLA biosynthesis. The decrease of enzyme activity may be attributed to the leakage of enzyme from the supports and denaturation of enzyme during the biocatalytic process over many reuse cycles (Liang et al., 2020).

It is noticed that the intrinsic fragile nature of enzymes makes them prone to denaturation or destabilization in harsh practical conditions, leading to unavoidably shortened lifespan and extremely high cost. Thus, we provided a possible synthesis strategy for application of enzyme immobilization by innovatively magnetic modifications to MAF-7. Herein, LDH/MNPs@MAF-7 exhibited more stable catalytic performances than those of free enzymes, including improved enzyme efficiency, stability and recyclability, due to the protection of enzymes by highly ordered frameworks. In addition, it exhibited excellent resistance to organic reagents, which makes it suitable for the industrial bioconversion of poorly soluble substrates. To summarize, the considerable universality and expansibility of this synthesis strategy would promise great potential in industrial applications of immobilized enzymes.

## 4 Conclusion

LDH and MNPs were successfully encapsulated into the MOF material MAF-7 by a one-pot coprecipitation strategy. The consequences of SEM, TGA, FT-IR, XRD, XPS, VSM and N_2_ adsorption characterization of LDH/MNPs@MAF-7 showed that LDH and Fe_3_O_4_ were successfully immobilized into MAF-7. The synthesized LDH/MNPs@MAF-7 particles exhibited stable catalytic activity in thermal stability, resistance to organic solvents and pH stability. What’s more, LDH/MNPs@MAF-7 exhibited good reusability, the residual activity remained above 80% after eight successive cycles of reuse. This study demonstrates that LDH/MNPs@MAF-7 could be a potential and magnetically recyclable biocatalyst for the manufactured production of D-PLA.

## Data Availability

The original contributions presented in the study are included in the article/Supplementary Material, further inquiries can be directed to the corresponding author.
