# Supplementary material for: One-pot encapsulation of lactate dehydrogenase and Fe3O4 nanoparticles into a metal–organic framework: A novel magnetic recyclable biocatalyst for the synthesis of D-phenyllactic acid
Source: Front Bioeng Biotechnol. 2023 Jan 9;10:1124450. doi: 10.3389/fbioe.2022.1124450 (PMC9868447; doi:10.3389/fbioe.2022.1124450)
Supplement: Supplementary file 1 [file Table1.DOCX]

Supplementary Material

One-pot encapsulation of lactate dehydrogenase and Fe_3_O_4_ nanoparticles into a metal–organic framework: A novel magnetic recyclable biocatalyst for the synthesis of D-phenyllactic acid

Xiaolong Sun, Jiahuan Hu, Yifeng Wang, Xi Luo, He Huang, and Yongqian Fu*

*** Correspondence:** Yongqian Fu: bioengineer@163.com

# Supplementary Experimental Section

# S 1.1 Expression and purification of D-lactate dehydrogenase

The expression and purification of D-LDH (abbreviated as LDH for convenience) were performed using the method from our previous study (Wang et al., 2022). The LDH expression strain was grown in Luria-Bertani (LB) medium containing 50 μg·mL^−1^ kanamycin at 180 rpm and 37 ℃ for 10 h. Then, the pre-culture was transferred to fresh LB/kanamycin medium for further growth at 37 ℃ and 180 rpm. After 2 h, lactose with a final concentration of 10 g·L^-1^ was added to induce expression, and the culture was continued for 12 h at 28 ℃ and 150 rpm. Cells were collected by centrifugation at 6300 g and 4 ℃ for 10 min, and LDH crude enzyme solution was obtained from lysed cells.

The LDH crude enzyme was loaded onto a Ni^2+^-nitrilotriacetic acid column (1.6 cm×10 cm, BioRad, USA) pre-equilibrated with buffer A (20 mM potassium phosphate buffer, pH 8.0, 500 mM NaCl and 20 mM imidazole). Buffer B (20 mM potassium phosphate buffer pH 8.0, 500 mM NaCl and 500 mM imidazole) was used to elute the LDH at a flow rate of 1.0 mL·min^-1^. The purified LDH was collected and dialyzed overnight against 20 mM potassium phosphate buffer (pH 8.0) to remove salt ions and imidazole.

# Supplementary Figures and Tables

## Supplementary Figures


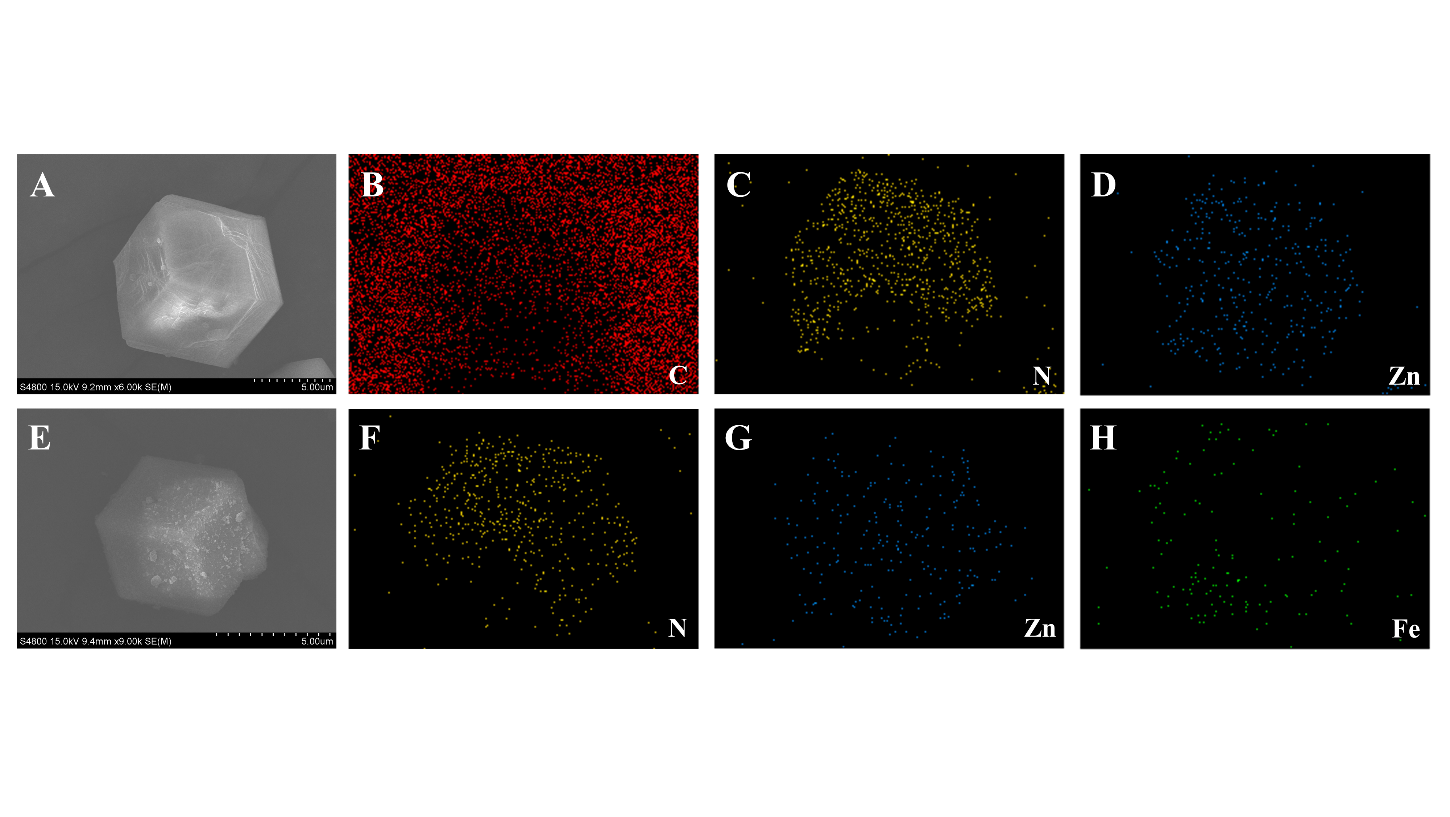


**Figure S1.** SEM spectra of MAF-7(A) and MNPs@MAF-7(E); elemental mapping of MAF-7(B-C) and MNPs@MAF-7(F-H)


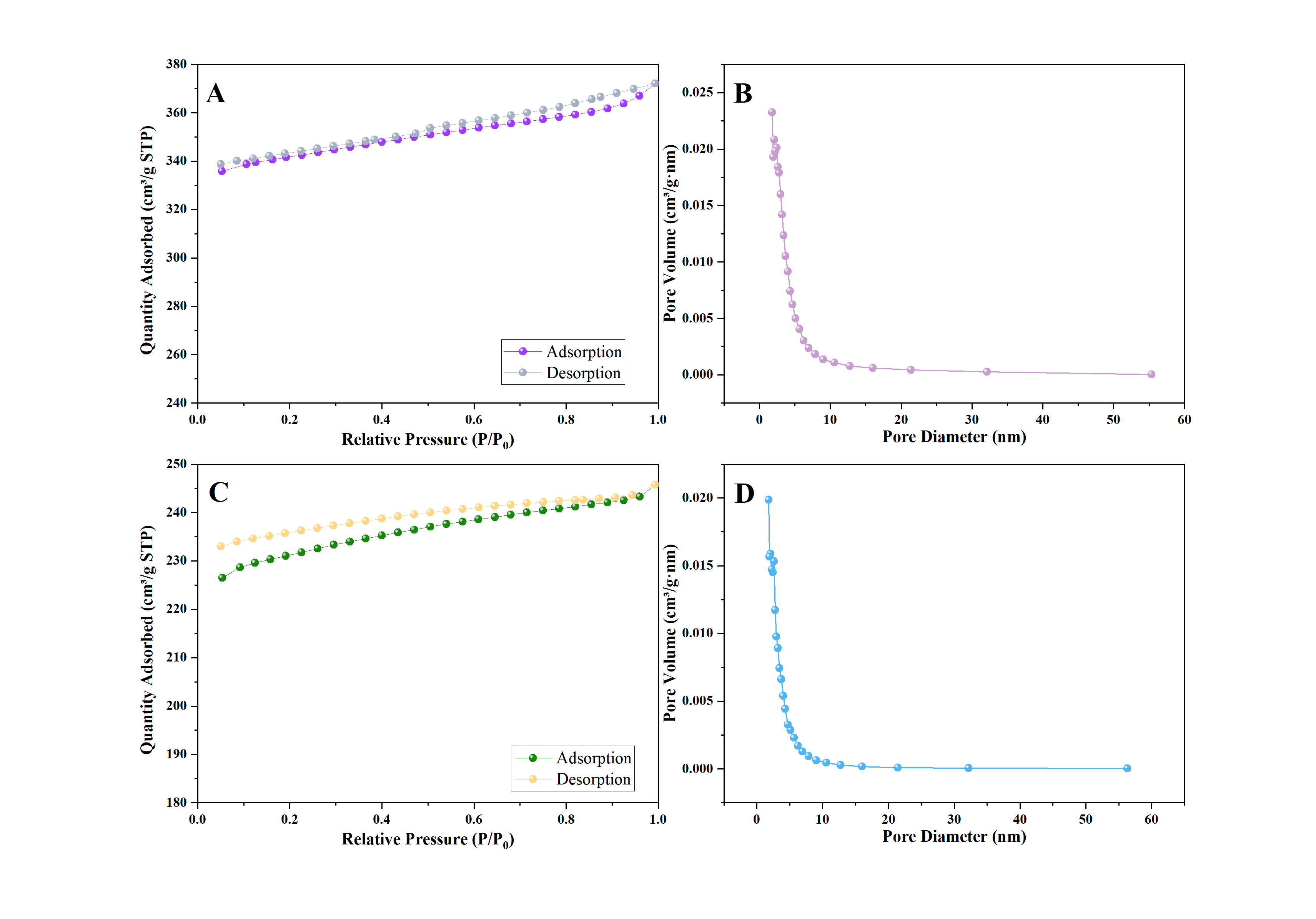


**Figure S2.** N_2_ adsorption-desorption isotherms of MAF-7(A) and MNPs@MAF-7(C); size distribution of MAF-7(B) and MNPs@MAF-7(D)


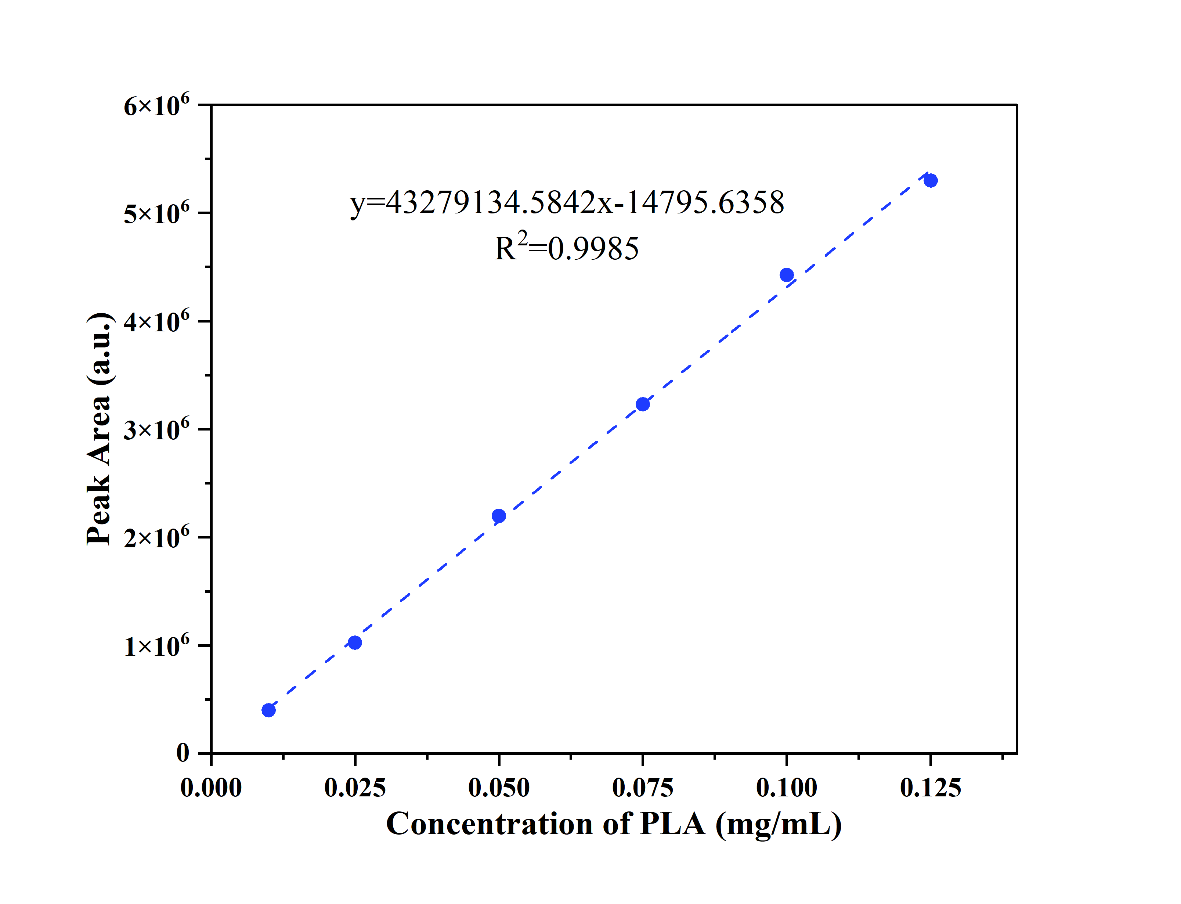


**Figure S3**. Standard curve of determination of enzyme activity


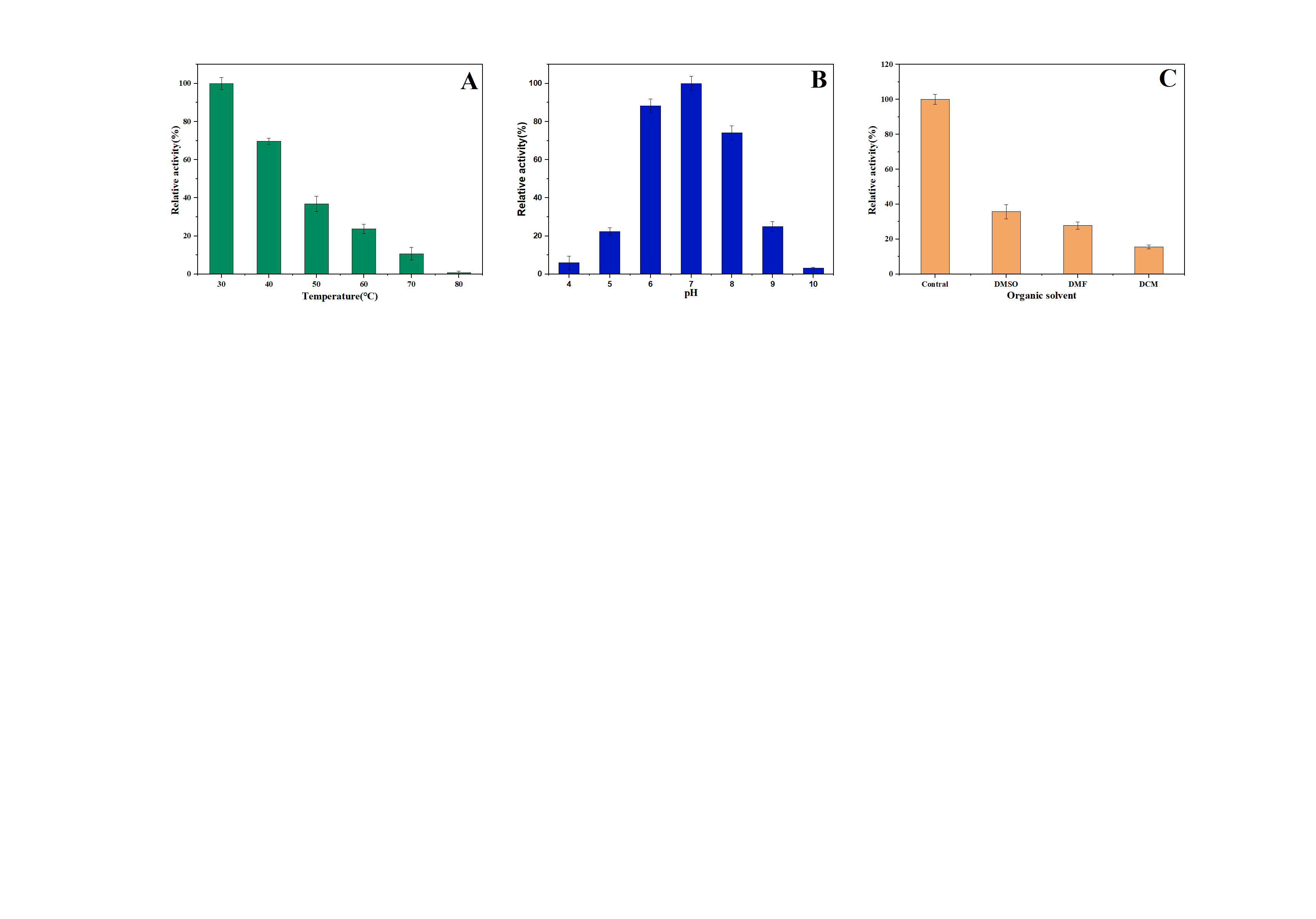


**Figure S4. (A):** Activity of LDH after incubation at the indicated temperatures for 20 min; (B): Activity of LDH after incubation at different pH values for 20 min; (C): Organic solvent stability of LDH

## Supplementary Tables

**Table S1.** Results of the BET surface area of MAF-7, MNPs@MAF-7 and LDH/MNPs@MAF-7

|  | **BET Surface Area/(cm^3^/g^-1^)** | **Adsorption average pore width (4V/A by BET)/(nm)** |
| --- | --- | --- |
| MAF-7 | 1115.0401 | 2.06 |
| MNPs@MAF-7 | 755.9212 | 2.01 |
| LDH/MNPs@MAF-7 | 511.4734 | 2.36 |

**Table S2.** Surface element ratios of LDH/MNPs@MAF-7

| **Element** | LDH/MNPs@MAF-7 | |
| --- | --- | --- |
|  | **Weight %** | **Atomic %** |
| **C** | 14.51 | 26.30 |
| **N** | 4.99 | 7.76 |
| **Zn** | 7.14 | 2.38 |
| **O** | 35.63 | 48.47 |
| **Fe** | 36.47 | 14.21 |
| **P** | 1.15 | 0.81 |
| **S** | 0.10 | 0.07 |

# Supplementary Reference

Wang, Y., Luo, X., Sun, X., Hu, J., Guo, Q., Shen, B., et al. (2022). Lactate dehydrogenase encapsulated in a metal-organic framework: A novel stable and reusable biocatalyst for the synthesis of D-phenyllactic acid. *Colloids Surf B Biointerfaces* 216**,** 112604. doi: 10.1016/j.colsurfb.2022.112604.
